# Costs of Inpatient Rehabilitation for Ischemic Stroke in Patients with Dementia: A Cohort Swedish Register-Based Study

**DOI:** 10.3233/JAD-190749

**Published:** 2020-02-04

**Authors:** Minh Tuan Hoang, Ingemar Kåreholt, Mia von Euler, Linus Jönsson, Lena von Koch, Maria Eriksdotter, Sara Garcia-Ptacek

**Affiliations:** a Center for Alzheimer Research, Division of Clinical Geriatrics, Department of Neurobiology, Care Sciences and Society, Karolinska Institutet, Huddinge, Sweden; b Aging Research Center (ARC), Karolinska Institutet and Stockholm University, Stockholm, Sweden; c Institute of Gerontology, School of Health and Welfare, Aging Research Network – Jönköping (ARN-J), Jönköping University, Jönköping, Sweden; d Karolinska Institutet, Departments of Clinical Science and Education, Södersjukhuset, and Medicine, Solna, Stockholm, Sweden; e Department of Clinical Pharmacology, Karolinska University Laboratory, Karolinska University Hospital, Stockholm, Sweden; f Division of Neurogeriatrics, Department of Neurobiology, Care Sciences and Society, Karolinska Institutet, Stockholm, Sweden; g Division of Family Medicine and Primary Care, Department of Neurobiology, Care Sciences and Society, Karolinska Institutet, Huddinge, Sweden; h Neuro Theme, Karolinska University Hospital, Stockholm, Sweden; i Aging Theme, Karolinska University Hospital, Stockholm, Sweden; j Section for Neurology, Department of Internal Medicine, Södersjukhuset, Stockholm, Sweden

**Keywords:** Cost analysis, dementia, rehabilitation, register studies, stroke, Sweden

## Abstract

**Background:**

Stroke and dementia are frequent comorbidities. Dementia possibly increases total costs of stroke care, especially cost of institutionalization and informal medical care. However, stroke rehabilitation costs in dementia patients are understudied.

**Objective:**

To estimate inpatient stroke rehabilitation costs for Swedish dementia patients in comparison with non-dementia patients.

**Methods:**

A longitudinal cohort study with linked data from the Swedish Dementia Register and the Swedish Stroke Register was conducted. Patients diagnosed with dementia who suffered a first ischemic stroke between 2010 and 2014 (*n* = 138) were compared with non-dementia patients (*n* = 935). Cost analyses were conducted from a Swedish health care perspective. The difference of rehabilitation costs between the two groups was examined via simple linear regression (before and after matching by propensity scores of dementia) and multiple linear regression.

**Results:**

Mean inpatient rehabilitation costs for dementia and non-dementia patients were SEK 103,693/$11,932 and SEK 130,057/$14,966, respectively (median SEK 92,183/$10,607 and SEK 106,365/$12,239) (*p* = 0.001). Dementia patients suffered from more comorbidities and experienced lower functioning, compared to non-dementia patients. The inpatient rehabilitation cost for patients with known dementia was 0.84 times the cost in non-dementia individuals.

**Conclusion:**

Dementia diagnosis was significantly associated with lower inpatient stroke rehabilitation costs. This might be explained by physicians’ beliefs on the limited effectiveness of rehabilitation in dementia patients. Further research on cost-effectiveness of stroke rehabilitation and patients’ satisfaction with stroke rehabilitation is necessary.

## INTRODUCTION

Stroke and dementia are frequently comorbid conditions. These two diseases are among the most common causes of death and disability in Sweden [1, 2]. The total cost of stroke to the Swedish society has been estimated at SEK 18.3 billion per year (around $2.1 billion) [3]. The societal costs of dementia in Sweden were estimated at SEK 62.9 billion (about $7.2 billion) [4].

Stroke rehabilitation is essential for recovery, also in individuals with dementia as they have a higher rate of disability and mortality [5]. The objective of stroke rehabilitation is to enable patients to relearn or redefine their body functions, become as independent as possible, and achieve the highest possible quality of life. In Sweden, most stroke patients admitted to the hospital receive acute care in a stroke unit. After the initial inpatient care, patients are discharged home, to inpatient rehabilitation, or to special accommodation (nursing homes or alternative solutions) arranged by the municipalities. Geriatric rehabilitation is assigned to patients aged 65 and above, while rehabilitation which includes return to work programs is offered to individuals < 65 [6, 7]. Outpatient rehabilitation can be delivered in the patients’ home by interprofessional teams, in outpatient clinics in primary health care or in the hospital, or by the municipality [6]. Because of a highly decentralized health care system, stroke care and rehabilitation in Sweden can be described in three levels: the state, the counties, and the municipalities [8, 9]. The organization of rehabilitation varies among counties in Sweden. The county councils administer specialized care (providing acute care, inpatient rehabilitation and outpatient rehabilitation for stroke patients at hospitals) and primary care (supplying primary, secondary prevention and long-term rehabilitation at primary care centers) [8, 9]. The municipalities take charge of home help services, special accommodation and share the responsibility for long-term rehabilitation with county councils in several regions [8, 9]. Private providers also participate in rehabilitation and home help services.

Stroke rehabilitation creates a high economic burden for society in the medium term, even if it potentially reduces long-term costs. Stroke rehabilitation amounted to roughly 40% of total costs for stroke care in Sweden [9–11]. Inpatient rehabilitation was the most expensive part of the first-year stroke care costs: rehabilitation amounted to 37% of these costs in Switzerland [12], 71% (together with acute care) in Denmark [13], and 89.5% (direct costs together with acute care) in Italy [14].

Dementia might influence stroke treatment and rehabilitation; hence, probably impacting stroke care costs. Previous studies found that dementia was associated with increased total costs of care, especially the cost of institutionalization and informal medical care [4, 15]. A study in Italy showed increased stroke care costs among patients with moderate and severe neurologic impairment [14].

Cost analyses of inpatient stroke rehabilitation for dementia patients are under-investigated. Dementia patients had higher direct medical costs only in the presence of co-morbidities in one study [16]. Hence, it is essential to evaluate the costs of stroke inpatient rehabilitation among dementia patients. Understanding cost drivers related to dementia in stroke rehabilitation could help policymakers optimize quality and efficiency of care and could help clinics understand the inpatient stroke rehabilitation costs for dementia and non-dementia patients. This study aimed to investigate costs of inpatient stroke rehabilitation among Swedish dementia patients in comparison with non-dementia controls.

## METHODS

### Study design and setting

This longitudinal cohort study was based on data originating from the linkage of the Swedish Dementia Registry (SveDem) and the Swedish Stroke Register (Riksstroke) through patients’ personal identification number. SveDem is a Swedish national quality register for dementia, including data on people diagnosed with dementia and annual follow-ups [17]. Patients are registered at the time of dementia diagnosis, including information about demographics, dementia categories, living situation, medication, and cognition levels [17]. The coverage is estimated to be 30–35% of all dementia patients in Sweden based on an estimated dementia incidence in the different regions of Sweden [18]. Riksstroke is a national quality database for stroke care in Sweden, which covers over 90% of strokes in Sweden [3, 19]. For each individual patient, data from before stroke onset, during the hospitalization and at discharge are collected [3, 19]. In addition, information at three-months follow-up is included, with a coverage rate of 80–90% [3, 19]. The Swedish National Patient Register, which encompasses all in-hospital and specialist diagnoses and surgical treatments [20], was employed to explore the comorbidities of participants.

### Participants and study size

**Fig.1:**
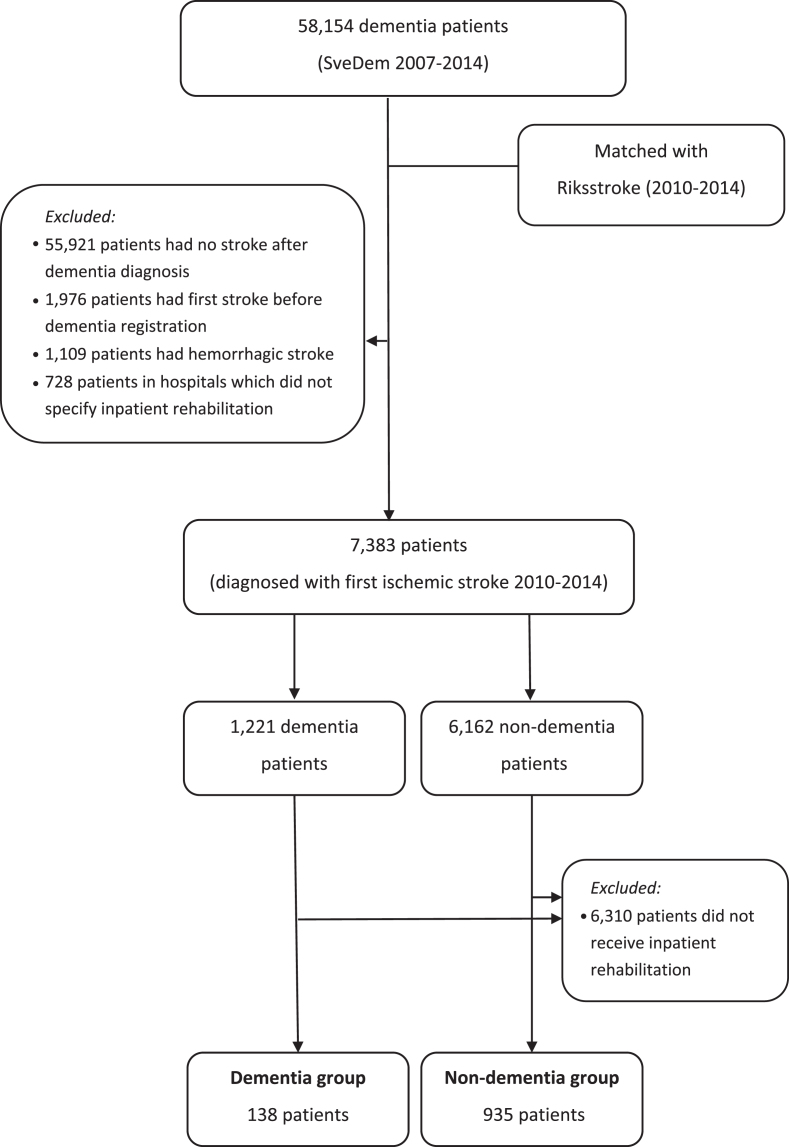
Patient selection

Data from 58,154 patients in SveDem between 2007 and 2014 were merged with Riksstroke between 2010 and 2014 to form two research groups (Fig. 1). The dementia group included SveDem patients who suffered from a first ischemic stroke after dementia diagnosis. The control group comprised non-dementia patients who experienced a first ischemic stroke. Non-dementia patients were defined as patients who were not registered in SveDem nor had a dementia or confusion diagnoses in the Swedish National Patient Register and did not use anti-dementia medications. Patients who suffered ischemic stroke before dementia diagnosis or hemorrhagic stroke at any time were excluded. In several hospitals, inpatient rehabilitation is included and indistinguishable from acute care. Therefore, patients in nine hospitals (out of 74 in total in our study) which did not specify inpatient rehabilitation were also excluded. This left 1,221 dementia patients and 6,162 controls (*n* = 7,383) included in the analyses of the likelihood of receiving inpatient stroke rehabilitation (Fig. 1).

The main analyses evaluated cost of inpatient rehabilitation; therefore, we excluded patients who did not receive inpatient stroke rehabilitation. The analyses on cost of inpatient rehabilitation encompassed 1,073 patients in the dementia group (*n* = 138) and the non-dementia group (*n* = 935) (Fig. 1).

### Cost calculation

The cost estimation was conducted from the Swedish health care perspective. Due to data availability, we only assessed direct costs of inpatient rehabilitation. This omitted informal care costs and outpatient rehabilitation costs. The cost generated from each patient was calculated by multiplying days in inpatient rehabilitation with average unit cost of inpatient stay. The length of stay in the rehabilitation unit was available in Riksstroke. The average cost per bed-day in a rehabilitation unit was SEK 6,932 in 2017, which originated from the cost per patient from the database of Swedish Municipalities and County Councils (Sveriges Kommuner och Landsting) [21]. This unit cost was converted to 2018 value (SEK 7,091) with inflation rate of 2.3% from the Swedish Consumer Price Index [22]. Costs were also converted into US Dollars based on the Sveriges Riksbank – Sweden’s Central Bank annual average conversion rate in 2018 ($1 = SEK 8.69) [23].

### Variables and data sources

The direct cost of inpatient stroke rehabilitation generated from each patient was the main outcome variable of the study. Independent variables were collected from the registers. Via SveDem, dementia status and sex of dementia patients were extracted. Comorbidities before stroke were obtained from the Swedish National Patient Register. Atrial fibrillation, diabetes, femur fracture, heart failure, and hypertension were selected for evaluation because of high prevalence among stroke patients or because they affect patient’s daily activities [24]. Other baseline variables and features during acute care were collected from the Riksstroke hospital-reported acute care protocols [19]. Three-month follow-up variables were extracted from Riksstroke’s patient-reported survey [19]. Living situation before stroke included: living at home without help, living at home with help, and living in an institution (special accommodation, acute care, or others) [19]. Primary activities in daily living (ADL) before and three months after stroke comprised clothing (without help/with help), mobility (without help, without help only indoors and needs help in- and outdoors), and toileting (without help/with help) [19]. Consciousness levels on admission to hospital were assessed via the Reaction Level Scale (RLS), which was categorized into three levels: fully awake (RLS 1), drowsy (RLS 2-3), and unconscious (RLS 4–8) [19, 25]. Complications during acute care, which include deep venous thrombosis, pulmonary embolism, fracture, and pneumonia, were also recorded. The length of stay in hospital was specified as days in acute care, in inpatient rehabilitation, and total days in hospital (including both acute care and inpatient rehabilitation stay). Place of discharge after acute care was classified into inpatient rehabilitation, home, special accommodation or other institution (such as acute clinical department, different stroke unit, etc.). Rehabilitation after discharge from inpatient rehabilitation, which was reported by patients three months after stroke, included home rehabilitation, day rehabilitation, special accommodation with rehabilitation, and no need for further rehabilitation. The modified Rankin Scale (mRS) is a clinician-reported measure of disability used for assessing functioning outcome after stroke [26, 27]. Higher mRS scores indicates worse functioning. Riksstroke has validated a conversion method to obtain mRS from the follow-up forms collected in the register [26]. Because it is impossible to distinguish between functioning with the mRS scores 0, 1, and 2 in Riksstroke, these scores were merged [26].

### Statistical methods

The association between dementia status and the likelihood of receiving inpatient rehabilitation (*n* = 7,383) was assessed by a binary logistic regression, adjusted with age at stroke, sex, living situation before stroke, consciousness levels at hospital admission, pre-stroke ADL, having complications during acute care, pre-stroke comorbidities, and length of acute care.

Attributes of 1,073 participants with inpatient rehabilitation was presented for three time points: 1) before stroke, 2) during acute care and right after hospital discharge, and 3) at three-month follow up. Numerical variables were summarized with means and standard deviation if normally distributed, or with median and interquartile range if not. Student *t*-test or Mann-Whitney U test was utilized to analyze the difference between dementia and non-dementia groups, as applicable. Categorical variables were presented as number of cases and percentages, and then examined by Pearson’s Chi square test.

The association between stroke inpatient rehabilitation cost and dementia status was investigated by linear regressions. Multiple linear regression and propensity score matching were employed to control for potential confounders, such as age at stroke, sex, living situation before stroke, consciousness at hospital admission, pre-stroke ADL and comorbidities, complications during acute care, and days in acute care. The selected variables were those identified through the literature review and our clinical practice, and those which presented differences between groups at the time points before stroke and during and right after acute care. Propensity scores were estimated by binary logistic regression with dementia/non-dementia as dependent variable and the above attributes as independent variables. Patients in the two groups were matched with 0.01 tolerance, which was appropriate to balance between getting a precise match (nearest-neighbor matching) and not dropping too many controls [28]. Finally, we obtained 138 dementia patients, matched with 125 non-dementia controls (810 unmatched controls were excluded). Another linear regression between cost and dementia status was then performed in the matched database. The linear regression after propensity score matching was also a sensitivity analysis to assess the impact of potential confounding factors on the results. A natural log transformation of the cost was applied to fix the violation of normal distribution in regression models.

SPSS version 25 (IBM Corporation, Armonk, NY, USA) was employed to perform the statistical analysis in this study. All statistical tests were two tailed with a *p*-value less than 0.05 considered statistically significant. Missing data were handled by excluding cases pair-wise.

### Ethical considerations

Patients are informed about registration in SveDem and Riksstroke at the time of dementia diagnosis or stroke hospitalization. They can refuse to participate in the register and also withdraw their data from the register at any time. For a research project, an approval from the regional ethics committee is needed. This study was approved by the Stockholm regional ethics committee including an approval that this study did not require individual consent from each participating patient (no. 2015/743-31/4). All information from the registries was anonymized and personal numbers were blinded to the researchers.

## RESULTS

### Characteristics of patients who did receive inpatient stroke rehabilitation (n = 1,073)

The baseline characteristics before stroke of 138 dementia and 935 non-dementia patients are presented in Table 1. There was no statistically significant difference in age or sex among the two groups. The level of independence with clothing, mobility, and toileting before stroke was lower in dementia patients compared with non-dementia controls (*p* = 0.001). Comorbidities were significantly more common in the dementia group compared to the non-dementia group regarding diabetes, femur fracture, and hypertension.

**Table 1:** Demographic characteristics, functioning, and comorbidities before stroke among patients receiving inpatient rehabilitation (*n* = 1,073)

	Dementia (*n* = 138)	Non-Dementia (*n* = 935)	*p*^a^
Age at stroke (y)^b^	83.2 (5.8)	82.5 (6.7)	0.257
Sex (men)	52 (37.7)	381 (40.7)	0.493
Living situation:
*home without help*	54 (39.1)	683 (73.0)	0.001
*home with help*	63 (45.7)	194 (20.8)
*institution*	21 (15.2)	55 (5.9)
*missing*	0 (0)	3 (0.3)
Clothing:
*without help*	103 (74.6)	877 (93.8)	0.001
*with help*	31 (22.5)	50 (5.4)
*missing*	4 (2.9)	8 (0.9)
Mobility:
*without help in-* & *outdoors*	104 (75.4)	867 (92.7)	0.001
*without help only indoors*	26 (18.8)	45 (4.8)
*with help*	6 (4.4)	16 (1.7)
*missing*	2 (1.4)	7 (0.8)
Toileting:
*without help*	115 (83.3)	888 (95.0)	0.001
*with help*	19 (13.8)	40 (4.3)
*missing*	4 (2.9)	7 (0.7)
Comorbidities:
*atrial fibrillation*	44 (31.9)	262 (28.0)	0.348
*diabetes*	34 (24.6)	165 (17.6)	0.049
*femur fracture*	21 (15.2)	83 (8.9)	0.019
*heart failure*	30 (21.7)	181 (19.4)	0.511
*hypertension*	84 (60.9)	469 (50.2)	0.019

Data presented as *n* (%) unless otherwise specified. ^a^The *p*-value comparing dementia and non-dementia group by Pearson Chi-square test (categorical variables) except age (using *t*-test). ^b^Years. Mean (standard deviation).

Consciousness levels at admission to hospital did not differ significantly between the groups (Table 2). During acute care, the number of patients who experienced complications was significantly different, with a proportion of less than 10% complications in both groups (Table 2). The median length of stay in acute care and inpatient rehabilitation in the dementia group were 6 and 13 days, respectively, compared to 6 and 15 in their non-dementia counterparts (*p* < 0.05). The need for further rehabilitation after discharge from inpatient rehabilitation differed significantly. Dementia patients reported more frequently that they did not need further rehabilitation (31.9%), compared to the counterparts (22.8%).

**Table 2:** Patients’ medical complications during acute care, length of hospitalization, rehabilitation after discharge from inpatient rehabilitation and costs (*n* = 1,073)

	Dementia (*n* = 138)	Non-Dementia (*n* = 935)	*p*
Consciousness at hospital admission:			0.275
*fully awake*	116 (84.1)	842 (90.0)
*drowsy*	18 (13.0)	84 (9.0)
*unconscious*	1 (0.7)	8 (0.9)
*missing*	3 (2.2)	1 (0.1)
Complications during acute care	6 (4.4)	66 (7.1)	0.017
*missing*	1 (0.7)	0 (0)
Total days in hospital^a^	18 (11)	22 (18)	0.001
Days in acute care^a^	6 (3)	6 (5)	0.018
Days in inpatient rehabilitation^a^	13 (10)	15 (14)	0.002
Rehabilitation after discharge^b^			0.001
*home rehabilitation*	29 (21.0)	315 (33.7)
*day rehabilitation*	6 (4.4)	103 (11.0)
*special accommodation with rehabilitation*	26 (18.8)	109 (11.7)
*no need*	44 (31.9)	213 (22.8)
*missing*	33 (23.9)	195 (20.8)
Cost of inpatient rehabilitation^a^:
*in SEK*	92,183 (67,364)	106,365 (99,274)	0.001
*in US*$	10,607 (7,752)	12,239 (11,424)

Categorical variables presented as *n* (%). Pearson Chi-square test performed, as appropriate. ^a^Numerical variables presented as median (interquartile range). Mann-Whitney U test was used because of skewed distribution. ^b^Three months after stroke, patients reported the types of rehabilitation/support that they had received after hospitalization. Their answers included: home rehabilitation, day rehabilitation, special accommodation with rehabilitation and “I did not need further rehabilitation or support”.

Three months after stroke onset, the proportion of dementia patients independent for clothing, mobility, and toileting was lower, compared to non-dementia groups (Table 3). Individuals with dementia were more disabled according to mRS scores. The percentage of patients with mRS scores greater than 2 was 84.1% among the dementia and 76.5% in the non-dementia groups.

**Table 3:** Patients’ functioning three months after stroke (*n* = 1,073)

	Dementia (*n* = 138)	Non-Dementia (*n* = 935)	*p*
Clothing:
*without help*	41 (29.7)	541 (57.9)	0.001
*with help*	67 (48.6)	276 (29.5)
*missing*	30 (21.7)	118 (12.6)
Mobility:
*without help in-* & *outdoors*	31 (22.4)	397 (42.5)	0.001
*without help only indoors*	28 (20.3)	241 (25.8)
*with help*	48 (34.8)	180 (19.2)
*missing*	31 (22.5)	117 (12.5)
Toileting:
*without help*	48 (34.8)	615 (65.8)	0.001
*with help*	62 (44.9)	203 (21.7)
*missing*	28 (20.3)	117 (12.5)
modified Rankin Scale:
*0-1-2*	5 (3.6)	158 (16.9)	0.001
*3*	33 (23.9)	366 (39.2)
*4*	30 (21.8)	174 (18.6)
*5*	40 (29.0)	113 (12.1)
*6*	13 (9.4)	62 (6.6)
*missing*	17 (12.3)	62 (6.6)

Data presented as *n* (%). Pearson Chi-square test was performed.

### Factors associated with receiving inpatient rehabilitation (n = 7,383)

**Fig.2:**
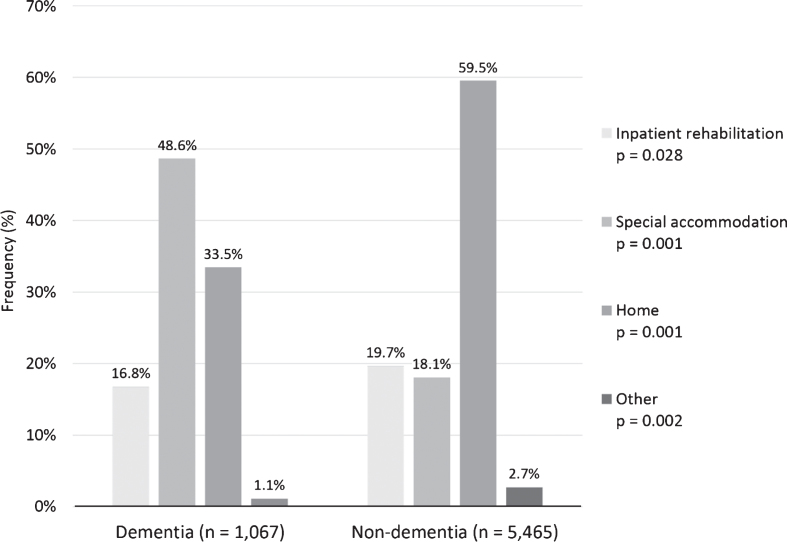
Discharge place after acute stroke care between dementia and non-dementia patients (*n* = 7,383; missing values *n* (%): dementia 154 (12.6), non-dementia 697 (11.3)). *P*-values for the difference between dementia and non-dementia patients.

Patients in the two groups differed significantly regarding where they were discharged after acute care (Fig. 2). A large proportion of patients in both groups were discharged home or to special accommodation after acute care. The percentage of patients discharged to inpatient rehabilitation was 16.8% and 19.7% in dementia groups and controls, respectively.

Dementia status was significantly associated with lower probability of receiving inpatient rehabilitation, as shown in Table 4. Individuals with dementia had 26% lower odds of receiving inpatient rehabilitation compared to non-dementia controls. However, when adjusting for demographic factors, living situation before stroke, consciousness on hospital arrival, pre-stroke ADL and comorbidities, complications during acute care and days in acute care, this difference was smaller and not significant. The likelihood of receiving inpatient rehabilitation was significantly associated with consciousness levels, mobility, complications, comorbidities, and days in acute care (Table 4).

**Table 4:** Odds ratio of receiving inpatient stroke rehabilitation in relationship with dementia status (*n* = 7,383)

	*Model 1 (n* = *7,383)*	*Model 2 (n* = *7,033)*
Dementia	0.74 (0.63 – 0.88)	0.90 (0.75 – 1.09)
Age at stroke		1.01 (1.00 – 1.02)
Sex (men)		0.89 (0.79 – 1.01)
Living situation before stroke:
*home without help*		reference
*home with help*		1.13 (0.86 – 1.48)
*institution*		1.30 (1.00 – 1.69)
Clothing before stroke:
*without help*		reference
*with help*		1.07 (0.73 – 1.57)
Mobility before stroke:
*without help in-* & *outdoors*		reference
*without help only indoors*		2.24 (1.34 – 3.73)
*with help*		1.54 (0.96 – 2.48)
Toileting before stroke:
*without help*		reference
*with help*		0.72 (0.46 – 1.13)
Comorbidities before stroke:
*atrial fibrillation*		1.09 (0.94 – 1.26)
*diabetes*		1.18 (1.00 – 1.38)
*femur fracture*		1.30 (1.05 – 1.62)
*heart failure*		1.20 (1.01 – 1.41)
*hypertension*		1.06 (0.93 – 1.20)
Consciousness at hospital admission:
*fully awake*		reference
*drowsy*		3.54 (2.10 – 5.94)
*unconscious*		3.29 (1.91 – 5.69)
Complications during acute care		2.14 (1.66 – 2.76)
Days in acute care		0.96 (0.96 – 0.97)

Data presented as odds ratio and 95% confidence interval. Model 1: Binary logistic regression model, unadjusted. Model 2: Binary logistic regression model, adjusted for age at stroke, sex, living situation before stroke, pre-stroke activities of daily living, pre-stroke comorbidities, consciousness at hospital admission, having complications during acute care, and number of days in acute care.

### Inpatient rehabilitation costs in relationship to dementia status (n = 1,073)

The average cost of inpatient rehabilitation was considerably lower in dementia patients compared to their non-dementia counterparts: mean SEK 103,693/$11,932 versus SEK 130,057/$14,966 (median SEK 92,183/$10,607 versus SEK 106,365/$12,239). There was a statistically significant difference in the cost of inpatient rehabilitation between the two groups (Table 2).

In the unadjusted simple linear regression model, dementia status was significantly associated with inpatient rehabilitation cost (Table 5). This corresponds to an inpatient rehabilitation cost for dementia patients 0.84 times of the cost in non-dementia patients.

**Table 5:** Inpatient stroke rehabilitation cost in relationship with dementia status (*n* = 1,073)

	*Model 1 (n* = *1,073)*	*Model 2 (n* = *1,056)*	*Model 3 (n* = *263)*
Dementia	0.84 (0.73 – 0.95)	0.86 (0.75 – 0.98)	0.76 (0.64 – 0.89)
Age at stroke		1.00 (0.99 – 1.01)
Sex (men)		0.93 (0.85 – 1.02)
Living situation before stroke:
*home without help*		reference
*home with help*		0.95 (0.84 – 1.06)
*institution*		0.79 (0.65 – 0.95)
Clothing before stroke:
*without help*		reference
*with help*		0.93 (0.69 – 1.24)
Mobility before stroke:
*without help in-* & *outdoors*		reference
*without help only indoors*		1.17 (0.98 – 1.40)
*with help*		0.84 (0.70 – 1.00)
Toileting before stroke:
*without help*		reference
*with help*		1.30 (0.95 – 1.79)
Comorbidities before stroke:
*atrial fibrillation*		1.06 (0.95 – 1.18)
*diabetes*		1.08 (0.96 – 1.22)
*femur fracture*		1.04 (0.89 – 1.21)
*heart failure*		0.96 (0.85 – 1.09)
*hypertension*		0.92 (0.84 – 1.01)
Consciousness at hospital admission:
*fully awake*		reference
*drowsy*		1.12 (0.97 – 1.29)
*unconscious*		0.90 (0.77 – 1.06)
Complications during acute care		1.30 (1.08 – 1.56)
Days in acute care		1.02 (1.01 – 1.03)

Data presented as ratios (e^β^) and 95% confidence interval. A natural logarithm transformation of the cost was applied to fix the violation of normal distribution in regression models. Then, exponentiation was performed to see the relationship between inpatient stroke rehabilitation cost and dementia in each regression model. Model 1: Simple linear regression model, unadjusted. Model 2: Multiple linear regression model, adjusted for age at stroke, sex, living situation before stroke, pre-stroke activities of daily living, pre-stroke comorbidities, consciousness at hospital admission, having complications during acute care and number of days in acute care. Model 3: Linear regression model (after implementing propensity score matching with the risk for dementia).

The multiple linear regression model and the simple linear regression adjusted by propensity scores confirmed the result, with costs in dementia patients 0.86 or 0.76 times the cost in controls, respectively for each model. Complications during acute care and days in acute care also showed a significant association with inpatient rehabilitation cost (Table 5).

## DISCUSSION

The aim of this study was to calculate the average inpatient stroke rehabilitation cost of patients with and without dementia. Patients with dementia before stroke had a lower stroke rehabilitation cost than those without dementia before stroke but the two groups had similar likelihood of receiving inpatient stroke rehabilitation. The mean cost was SEK 103,693/$11,932 for patients with a dementia diagnosis compared with SEK 130,057/$14,966 in the non-dementia group (median SEK 92,183/$10,607 vs SEK 106,365/$12,239, *p* = 0.001). All linear regression models showed dementia diagnosis to be significantly associated with lower inpatient rehabilitation cost (Table 5).

The inpatient stroke rehabilitation cost for both dementia and non-dementia groups in Sweden was different from the cost in other European countries. In Denmark, the acute care and inpatient rehabilitation lasted 27 days on average and generated a mean direct cost of about SEK 174,860/$20,122 in 2018 value, although the study was published in the 1990s and results may not be comparable with current practices [29]. In Switzerland, inpatient rehabilitation lasted 39 days on average and generated a mean cost of about SEK 238,790/$27,479 (median SEK 198,218/$22,810) in 2018 value (37% of first-year stroke care costs) [12]. Another study indicated that acute care and inpatient rehabilitation costs were SEK 70,770/$8,144 in 2018 value, however, accounted for 89.5% of first-year stroke care direct costs in Italy [14]. In our study, the inpatient rehabilitation cost accounted for about 40% of the first-year costs, which were estimated in a previous Swedish population study [11]. The median length of inpatient rehabilitation for dementia patients was 13 days. These findings can be partially explained by different stroke care services among countries, different methods among studies, and the trend toward decreasing length of stay in stroke units in the past 20 years. Furthermore, the difference among Swedish patients might reflect the preference for home rehabilitation. Our study indicated that more than 80% of patients were assigned home rehabilitation or special accommodation after acute care (Fig. 2). Approximately 16–18% patients in both groups received inpatient rehabilitation after acute treatment. Additionally, early supported discharge with stroke rehabilitation at home in Sweden is now recommended by the Swedish National Guidelines for Stroke Care for stroke patients with mild and moderate symptoms [30]. However, this policy change is relatively recent and would not have been so widely implemented at the time of our study. Previous studies also demonstrated that early hospital discharge with home rehabilitation not only decreases dependency, mortality, admissions to institutional care, and length of hospital stay [31, 32]; but also renders similar health benefits as conventional rehabilitation [33]. However, studies on early supported discharge have been limited to patients with mild to moderate disability after stroke and excluded patients with cognitive impairment, so the benefits of this intervention in patients with dementia or severe disability are unknown [31–33].

Patients with dementia prior to the stroke had significantly lower cost for inpatient stroke rehabilitation compared to their non-dementia counterparts. Before stroke, there was no statistically significant difference in age at stroke, sex, or level of consciousness at hospital admission between the two groups. Notwithstanding, dementia patients differed significantly from non-dementia controls in pre- and post-stroke basic ADL, and mRS three-months after stroke. The higher burden of disability in dementia patients compared to non-dementia patients was also identified in previous studies [5, 34]. Dementia diagnosis was not significantly associated with a lower likelihood of receiving inpatient rehabilitation after adjusting for functioning level and other attributes (Table 4). Poorer functioning, physical health, and lower inpatient rehabilitation cost occurred in the dementia group. This result is contrary to popular beliefs and a previous study in which dementia accounted for higher direct medical costs in the presence of comorbid conditions [16]. Several former studies also provided opposing evidence to our study, arguing that more pronounced disability accounted for higher stroke rehabilitation costs or inpatient care costs [8, 13, 14, 35–37].

One interpretation is that health care expenditure was allocated unequally to the more vulnerable population. This might be caused by health care professionals’ beliefs, who decide when and where patients should be discharged. First, health care professionals might infer lack of rehabilitation potential in patients with dementia, possibly discharging dementia patients earlier to primary care centers or municipalities’ special accommodation. Second, this contradiction might be justified by the similarities in rehabilitation of stroke care and dementia care. The Swedish National Guidelines for Dementia Care recommend cognitive and physical training for ADL in dementia patients [38]. Hence, physicians possibly reduce the length of inpatient stroke rehabilitation because dementia patients are discharged to a special nursing home for dementia care or primary centers, where they received dementia care before stroke, to resume training to maintain their ADL-functions. Physicians may deem this adequate also as stroke rehabilitation. A previous study by our group hypothesized that dementia patients could have returned to their previous residence, meanwhile non-dementia patients might have longer hospital length of stay waiting for a nursing home bed, or for home adaptations and home help to be in place [39].

Our study shows differences in health costs and resource allocation for stroke rehabilitation among dementia patients, in comparison with general stroke patients. Even though all Swedish residents are covered by a national health insurance and health care and rehabilitation costs are publicly funded, health inequality in stroke care and rehabilitation in Sweden have been shown in several previous studies [6, 39, 40]. The disparity in the provision of inpatient rehabilitation has implications for public health planning. The latest Swedish National Guidelines for Stroke Care recommend early supported discharge with coordinated rehabilitation at home in patients with mild to moderate symptoms [30]. However, it is unclear how much this policy influenced outcomes in our study, since it is meant as a guideline for patients with mild and moderate disability only [31–33].

Without more information, it is impossible to ascertain whether dementia patients would have benefited from longer rehabilitation stays. Hence, further research on cost-effectiveness of stroke inpatient rehabilitation and home-based rehabilitation among dementia patients should be performed to assess their efficiency and resource allocation. Dementia patients’ expectation and satisfaction with inpatient and home-based rehabilitation should also be explored. It is necessary to investigate physicians’ beliefs that underlie these decisions.

To our knowledge, this is the first study to explore the inpatient rehabilitation cost in dementia patients, in comparison with non-dementia controls. A major strength of our study is the combination of various national databases enabling rigorous analyses on characteristics of stroke and dementia patients. A large, national cohort of dementia and stroke patients, and excellent coverage of Riksstroke are additional strengths of this study. Despite an increasing coverage rate through the years, SveDem captures about 30–35% of all estimated incident dementia patients in Sweden [18]. However, the actual incidence of dementia as diagnosed in regular clinical practice is probably lower, potentially leading to higher coverage for SveDem. We tried to overcome this obstacle by linking SveDem with other national registers, such as the Swedish National Patient Register and the drug register, to exclude patients with dementia diagnoses or those taking dementia medications from the control group. Extrapolation to other settings should be performed with caution because these findings represent the Swedish dementia population. As a register-based study, our research also faced certain limitations regarding incomplete data. For instance, patients’ pre-stroke functioning were only assessed by independence in clothing, mobility, and toileting. The mRS scores were exclusively available after stroke event. Therefore, the ADL functioning levels of patients before stroke were evaluated only to some extent and changes in mRS scores could not be ascertained. Stroke severity (for example measured with the National Institutes of Health Stroke Scale NIHSS) was not considered because of a large proportion of missing data for this scale in this sample subset. This could affect the results of the study because the NIHSS has an impact on the length of stay and rehabilitation cost. Another major weakness of this study was that results of the study reflect the costs of a specific type of stroke rehabilitation—inpatient rehabilitation. Calculating outpatient rehabilitation and indirect medical cost was not possible due to the unavailability of the data in the registers. It possibly leads to the underestimation of actual rehabilitation costs, although indirect and home care cost might possibly be higher. Another limitation is that many of the stroke and dementia patients were cognitively and physically impaired; hence, questions on their current health status and functioning were probably answered by their relatives or nurses and recall bias could be a factor. This might cause specific systematic errors in the study, particularly due to differences in cognitive impairment between the dementia and non-dementia groups. Missing information on genotypes and biomarkers was also a weakness of our study. Genotypes or biomarkers are not available in our registers, which are based on clinical data obtained in normal clinical practice. However, to our knowledge, there are no studies reporting that genotypes or biomarkers are used or provide explanations for the choice of rehabilitation trajectory after stroke or the success of various ways of organizing rehabilitation. It is plausible that there might be important associations (for instance, the severity of the stroke might be associated with bio-markers, but stroke severity was adjusted for in the analysis). Alzheimer’s disease pathology and pathology for other types of dementia were not available in SveDem as well. Dementia diagnoses were conducted following regular clinical practice and clinical criteria. Our study reflected the clinical reality of stroke rehabilitation in patients with dementia in Sweden and, as in clinical practice, these diagnoses were mostly clinical. Last but not least, organization of stroke rehabilitation differs in Sweden. The criteria for inpatient stroke rehabilitation admission vary between the different hospitals but reflect the clinical reality of stroke rehabilitation in Sweden. According to the Riksstroke annual reports, the admission into inpatient rehabilitation depends on the functioning of the patient, the living conditions of the patient before stroke and the availability of hospital beds. In most of the smaller hospitals in Sweden, inpatient rehabilitation is integrated into acute care services which is not the case in the larger hospitals, particularly in urban areas. Therefore, in some hospitals inpatient rehabilitation days were reported as days in acute care. 728 patients in these nine ambiguous hospitals (out of 74 hospitals in total) were omitted from our analyses. This exclusion may distort the rehabilitation cost to some extent.

## CONCLUSIONS

Dementia status was significantly associated with inpatient stroke rehabilitation cost. The inpatient rehabilitation cost was lower in dementia patients compared to non-dementia patients due to a shorter rehabilitation stay. Dementia patients experienced lower functioning both before and after stroke. Future studies on stroke rehabilitation for dementia patients should investigate effectiveness of rehabilitation in this population, physicians’ behaviors and beliefs, as well as patients’ expectations on inpatient and home-based rehabilitation. Moreover, cost-effectiveness analysis of inpatient and home-based rehabilitation should also be performed.
